# Navigating authoritarian politics: towards reflexive framing in healthcare research

**DOI:** 10.1186/s12992-025-01115-6

**Published:** 2025-04-17

**Authors:** Marit Tolo Østebø, Kenneth Maes, Gabrielle Gibb, Rebecca Henderson

**Affiliations:** 1 Department of Anthropology, University of Florida, Gainesville, FL 32603 USA; 2 School of Language, Oregon State University, Culture & SocietyWaldo Hall 228, 2250 SW Jefferson Way, Corvallis, OR 97331 USA; 3 Newport Beach, USA

**Keywords:** Political reflexivity, Authoritarianism, Framing, Health-care research, Politics, Hidden curriculum

## Abstract

**Background:**

How do Northern Global Health scholars navigate authoritarian political contexts in their research in other countries? This question motivated the research project on which this article is based. Over ten months, we conducted in-depth qualitative interviews with sixteen European and North American scholars who were engaged in health-related research in an authoritarian country we refer to as Patria.

**Results:**

All our interviewees recognized health as a political matter and acknowledged the importance of considering politics in Global Health research. Yet, they were reluctant to explicitly integrate politically sensitive topics and discuss questions related to local political context in their research. To gain and maintain access, and to protect themselves and their local collaborators in a politically sensitive and authoritarian context, the researchers employed practices of ‘framing’. Such strategies included avoiding terms, scholarly references, and questions that were politically loaded; strategically conforming to the assumed apolitical language and methodologies of health research, and negotiating with and leaning on their local counterparts in processes of research dissemination and writing.

**Conclusion:**

Drawing on frame theory and literature on fieldwork and authoritarianism we discuss the implications our findings have, not only for Global Health research, but for healthcare sciences more broadly. While researchers who work in authoritarian regimes may be particularly prone to engage in practices of framing, the strategies our interviewees used are not limited to Global Health researchers working in such settings. As anthropologists with experience researching health in multiple countries, including in the United States, we recognize the strategies that our interlocutors used from our own research. By including a discussion of some of the ways political factors have shaped our research we make an argument for the value of *political reflexivity* in health research: the critical scrutiny of the taken-for-granted presuppositions and norms that guide our research, and of the political environments and power dynamics that shape and are shaped by our research. A turn to political reflexivity in health research can unravel some of the tacit assumptions, biases, norms and practices that are integral to the health care sciences and which students and researchers must critically think about.

## Background

How do Northern Global Health scholars navigate authoritarian political contexts in their research in other countries? This question motivated the research project on which this article is based. Over ten months, we conducted in-depth qualitative interviews with sixteen European and North American scholars engaged in health-related research in an authoritarian country in the Global South that we refer to as Patria (for reasons clarified below). Drawing on these interviews this article has two main aims: first, to argue for the importance of *political reflexivity* not only in Global Health research, but across the healthcare sciences more broadly. In doing so, we contribute to a growing body of literature that emphasizes the value of reflexivity and increased focus on power and politics in health research [27, 33, 43, 47, 51, 54, 57]. Our second aim is pedagogical: by showcasing how Global Health researchers, including ourselves, use practices of framing to navigate political processes, pressures and constraints, we seek to articulate parts of the “hidden curriculum” [29] in health research, so that it can become an explicit component in researcher training.

The relationship between health and politics has been much discussed in fields such as medical anthropology [28, 55], medical sociology [19], and political science [14, 25]. While a growing body of literature on the social – and even more so political – determinants of health pay attention to the impact political ideologies and systems have on health care outcomes and health systems [15, 18, 34], the relationship between politics and health remains neglected in much healthcare research. In many scholarly publications, health interventions and systems appear purely technical and scientific – as apolitical, detached from political systems and ideologies, and aloof from the everyday messy context of political contestation. The Covid-19 pandemic reminded us, however, that health and politics are deeply intertwined. In many countries, such as in the US, the pandemic and the public health response were highly politicized, fueling political polarization and resistance, and increasing distrust between governments and citizens [31].

Scholars who have critically discussed the lack of attention to political context and practices of power in health research commonly attribute this silencing to the hegemonic status of biomedicine. Some have argued that since health science professionals are mainly trained in medicine and biology, and not in the social sciences, they are not exposed to interdisciplinarity and do not have the necessary skills to understand political context and analyze complex power relations [47]. Others have suggested that health researchers depoliticize “unconsciously” [49] or that they disparage politics because they consider it to be a “dirty,” unsavory, and unscientific business that interferes with rational decision making [34, 37]. Drawing on the works of James Ferguson [21] some have also argued that the field of Global Health in particular acts as an “anti-politics machine” [10, 30, 42] – an apparatus which “denies ‘politics’ (…) everywhere whisking political realities out of sight” ([21], xv).

In this article, we examine the extent to which our interviewees, all Northern researchers with varying educational backgrounds, recognize health as a political matter and acknowledge the importance of considering politics in health research. We probe their awareness of the authoritarian nature of the Patrian government, and their willingness to explicitly integrate politically sensitive topics and discuss questions related to local political context in their research and publications. By detailing and discussing the strategic and unconscious practices of ‘framing’ [3, 22, 59] our interviewees employed as they navigated a challenging and authoritarian political context, we seek to add nuance to the broad-brushed characterization of Global Health as an anti-politics machine and of healthcare researchers as inattentive to politics.

Researchers working in authoritarian regimes, where the risks associated with conducting critical research often are higher than in other contexts, may be particularly inclined to engage in the framing practices we discuss in this paper. However, it would be a mistake to assume that the strategies our interviewees described are unique to health researchers working in such settings or limited to the power dynamics and politics that are at play in authoritarian regimes in the Global South. As anthropologists with experience researching health systems and socially engineered development in multiple countries, including in the United States, we recognize many of these strategies from our own research. With the increased political polarization and politicization of the health care sciences in many countries – particularly in countries in the Global North – and the subsequent increase in the vulnerability of healthcare researchers, it is likely that many scholars, consciously or unconsciously, employ the framing practices we detail here to mitigate political consequences. It is therefore our hope that this article can provide an entry point for a critical discussion of how power and politics play out within the healthcare sciences more broadly, and that it can lead to a deeper recognition of the fact that we always must negotiate with powerful actors in multiple arenas, including funders, international agencies, governments, leaders in our research locations, our research institutions, and academic publishers. As healthcare researchers we are, moreover, often firmly situated within these powerful structures ourselves.

The multiple ways that politics matter, and the questions political processes and power struggles pose to our research are, however, rarely explicitly dealt with in our publications or trainings, and warrant our second, pedagogical aim: by discussing some of the ways politics have shaped our research – including the writing and publication of this text – we hope to unravel tacit assumptions, norms and practices that are integral to the healthcare sciences and which students and researchers must learn and critically think about.

In the next section we present our analytical framework and situate ourselves within the larger frame theory tradition [5, 7, 24, 35]. We then introduce the study context, detail our methodology, and explain why we decided to fictionalize the geographical location that inspired our original research idea and questions. In the third section we detail how the researchers we interviewed navigate a politically challenging context and discuss these practices drawing on frame theory and the emerging literature on fieldwork and authoritarianism [23, 26]. Finally, we summarize and discuss the implications our findings have for Global Health research and practice and for the healthcare sciences more broadly, highlighting the value of political reflexivity.

## Frame theory

Framing, a term first used by Gregory Bateson [6, 24], is a relatively flexible label for a variety of approaches that seek to explain how we make sense of and publicly portray a particular issue or situation. Frame theory has been well developed within policy studies [7, 35, 59], communication studies [12], and in social movement theory [32], where it often is used to denote practices that actors utilize to influence political decision making or political debates. In more recent years, there has been a growing body of literature that employs frame theory within health research [5, 35, 46]. For example, Koon et al. provide an excellent overview of processes of framing research in the health sector [35]. Moreover, in a recent Lancet article, Shiffman and Shawar reviewed scholarship on Global Health policy making, examining how processes of framing shape Global Health priorities [54].

There are considerable variations in terms of how the existing literature on framing views the role of the human subject. Social movement theorists often portray framing as an intentional, conscious, and strategic process [7]. While we in the writing up of this article consciously employ strategic framing as a methodological and analytical tool, we also draw upon scholars who emphasize framing as an unintentional, discursive process [3, 40, 58, 60]. Inspired by Michel Foucault, a grounding premise of this intellectual tradition is that concepts are contested and open to multiple interpretations. Such an approach implies critical scrutiny of the assumptions that underpin the framing practices; that is, the unspoken and hidden elements that the dominant discourse is muffling, and the things that are silenced or excluded from the frame. By adopting a dual approach that recognizes the strategic as well as the unconscious aspects of framing practices we seek to “balance structure and agency” ([36], 2). We are particularly influenced by the works of the political scientist Carol Bacchi. While her analytical framework *What’s the Problem Represented to Be?* (WPRB) has been widely adopted as a tool for analyzing policy [11, 13, 16, 50], her discussion of practices of framing in relation to research, and the importance of recognizing its political nature [3, 4], may be less known. According to Bacchi, “research is an active component in the shaping of different realities and therefore is, at its core, a political practice” ([4], 142). Hence, it is essential that we as researchers engage in “reflexive framing” ([3], 19 ff.). Drawing on Bacchi and debates about reflexivity in anthropology [48, 61], we therefore make an argument for the value of “political reflexivity” ([61], 34 ff.), which we here define as the critical scrutiny of the taken-for-granted presuppositions and norms that guide our research, and of their relationships to the political environments and power dynamics in specific research locations as well as in academic research more broadly.

In analyzing our findings and in concurrence with our theoretical framework, we move beyond a narrow conceptualization of the political, in which politics are limited to the role of government, membership in political parties, and political ideologies and systems. By adopting a broad conceptualization of what constitutes governance and the political (see e.g. [3, 17], we link politics to power, priority setting, influence and involvement in policy formulation, and implementation and access to resources [27]. Such a broad interpretation of what constitutes the political allows for a recognition of the multiple arenas in which politics matter – ranging from powerful donor organizations to national governments, non-governmental organizations, research institutions, and academic journals. It also implies an acknowledgement of academics as political subjects [4].

## Study context and methodological considerations

For reasons detailed below, we have chosen to de-identify the country that was the focus of our research, using the pseudonym Patria. In what follows we carefully frame the study context in a way that can be descriptive of many authoritarian regimes.

Patria is located in the Global South and, alongside a history of attracting significant humanitarian attention, has a long-standing tradition of authoritarianism. Political power has historically been concentrated in the hands of a single leader or an ethnic group, marked by the suppression of dissent, restrictions on freedom of speech, and the use of coercion to maintain power and enforce policies. Religion, ethnicity, and economic inequality are politically sensitive issues that have fueled political instability, conflict and violence. While certain health topics – such as reproductive health and rights, and the role of Community Health Workers (CHWs) – may be more politically sensitive than others, in Patria, as in many similar regimes, political sensitivity is not limited to specific topics. Anything perceived as challenging the regime’s dominant narrative or political order can be deemed politically sensitive. For instance, failing to comply with seemingly arbitrary political demands – such as refusing to plant a mango tree in an area unsuitable for such plants – may be interpreted as a political act.

### De-contextualizing our research

Our decision to conceal the geographical location that inspired this research in the first place is partly informed by the feedback and rejections we have received from journal editors and external reviewers on earlier versions of this manuscript. In the first version submitted to a highly regarded health and social science journal, we named the country, described its historical, geographical, and political context in detail, and discussed the authoritarian nature of the regime. While two of the reviewers provided constructive comments and recommended ‘revise and resubmit’, the third concluded that our paper should be rejected – which it was. This third reviewer claimed that by referencing researchers who had been critical of the current regime in Patria, our paper was biased. We could perhaps say that the reviewer acted as “an intellectual police” ([62], 1463), a term that has been used to describe scholars who work to maintain the official and oppressive narratives and practices of authoritarian regimes. In our view, the review confirmed the very argument we were attempting to make: that health research is far from apolitical, and that we must recognize and critically reflect on the impacts that politics and political systems have on our research practices (including our reviews), as well as the potential political impacts our research, once published, could have. This leads us to our next point.

“Does publishing this paper risk the wellbeing of those who do health research in ‘Patria’? If publishing an article like this leads to increased scrutiny of health research by the regime, it is simply not worth it”. These comments from another critical reviewer highlight some of the ethical challenges associated with doing research in authoritarian settings [23, 26, 62], and also influenced our decision to use a fictive country name. While the renaming of individual persons or institutions, as a “protective practice” [23] is common in qualitative and anthropological research, such practices are less common when it comes to single countries. In this particular case, anonymization of the research context addresses the ethical concerns raised by the reviewer above redirecting our research to broader questions about how political and institutional context, and relations of power impact healthcare research more broadly.

### Study population and data collection

Our interest in exploring how Northern Global Health researchers engage with politics and navigate political context in an authoritarian country in the Global South was inspired by conversations we had with colleagues, and by our own experiences working and conducting research in authoritarian and conflict-prone countries. When we initiated the study, we had access to a systematic review showing that Global Health research conducted in one of these countries rarely discussed or considered local politics. While this absence indicates that politics and political determinants of health receive limited attention in Global Health publications, it does not reveal much about what researchers think and know about the relationship between health and politics, or how they navigate local political contexts. To better understand why politics were absent or excluded from publications pertaining to health in countries such as Patria, we designed a research project focusing on Northern Global Health researchers as our study population.

Over a period of ten months, we conducted semi-structured qualitative interviews with sixteen European and North American scholars. In addition to drawing on our own networks, we reached out to researchers that we, as part of reviewing and reading health related literature related to Patria, had encountered. We also identified informants using snow-ball sampling. Our sample included a broad group of researchers. More than half of our interviewees were trained in the health sciences, e.g. medicine (5), nursing (3), public health (1), and psychology (1), some of whom also had degrees (including PhDs) in more social-science oriented fields. The remaining interviewees were firmly situated in medical anthropology (4), history (1), and political science (1). Common for all the researchers was that they had been or still were engaged in and had published research pertaining to health and healthcare in Patria.

Prior to conducting the interviews, we developed a semi-structured interview guide seeking to understand how our research participants engaged with politics in their research. Many of the questions also addressed the impact the political context in Patria had on the research process and the kind of questions the researchers asked. We did not provide a definition of the term ‘political’, but left it open to the interviewees to respond according to their interpretations. Many of our interviewees responded to our broadly formulated questions by talking about the authoritarian nature, or the modes of governance of the Patrian regime. Except for one interview, which was done in person, we did the interviews using digital platforms such as Skype and Zoom.

### Why focus on northern global health researchers?

From a decolonizing Global Health perspective, one may ask – as previous reviewers of this manuscript have done – why we excluded researchers from Patria in our study. Our decision to only interview Northern Global Health researchers was partly influenced by the political situation in the country. We carried out this research remotely, without external funding. Since the Patrian government is known for its sophisticated digital surveillance system, we deemed it too risky to carry out interviews about sensitive political topics over the Internet. University politics at one of our home institutions also influenced our decision. A few months prior to initiating the study, one of the authors had asked that the Institutional Review Board (IRB) at their university waive the requirement for a local research permit for another politically sensitive project in Patria. They argued that a research permit request through official channels in Patria potentially could pose a danger to informants. As others who have conducted work in authoritarian contexts also have argued, such a request “would only serve to attract the authorities’ attention (…), arouse suspicion, and most likely result in a denial” ([23], 20). The aforementioned university IRB refused to waive the requirement for a local permit, and this experience further discouraged us from including researchers from Patria.

Our rationale for examining Northern Global Health researchers was also inspired by an interest in examining how researchers, privileged by their connections to higher institutions in the Global North, and their European and North American citizenships, navigate the politics of health research in Patria. In other words, we wanted to engage in a more self-critical discussion with a group to which we ourselves belong. Drawing on Seye Abimbola’s reflection on the local vs. foreign gaze in Global Health, we could perhaps say that our position and our gaze may be viewed as “ideal”: since we as ‘insiders’ and members of the Global Health research community are “local people writing about local issues for a local audience” ([1], 2). This does not mean that we claim that the position and the gaze we apply in this particular research project provides a comprehensive picture of the politics of Global Health research in a context like Patria, or that we think the perspectives of researchers from Patria are unimportant. Our point is rather to highlight that the category Northern Global Health researcher, as a privileged study population within the larger Global Health ecosystem, is worth critical scrutiny. In retrospect, we acknowledge that we could have explored other ways in which to include researchers from Patria in this project. For instance, we could have invited them as collaborators or co-authors, still interviewing Northern researchers. While such collaboration might have posed ethical challenges—such as risks to their current or future employment—we could have responsibly co-developed strategies to mitigate these responsibly. We also recognize the importance of capturing the experiences of researchers from the Global South conducting research in authoritarian contexts within the Global North. These limitations in our project lead to clear recommendations for future studies that compare and contrast political reflexivity – or its absence – in health research conducted across the Global North and South.

## Results

### Engagement with politics

All the researchers we interviewed, regardless of educational background, recognized health as a political matter, and acknowledged the importance of considering political context in health research. They were – generally speaking – aware of the authoritarian nature of the Patrian government. The extent to which the researchers were informed of the ways that practices of authoritarianism played out on the ground varied considerably, however. A few of our interviewees had in-depth knowledge about local political dynamics and were very aware of the regime’s authoritarian practices. They would talk about the regime and the increase in human rights violations, such as the arbitrary jailing and disappearance of opposition leaders, journalists, and others who opposed or questioned the official government narrative. They were cognizant of the restrictions on freedom of speech and of the government’s detailed network of informants tasked with reporting on the behaviors of their friends and neighbors. They also critically reflected on the potential ramifications these practices had on the health system and on their research. The majority of the researchers we interviewed, however, had limited knowledge of these dynamics.

As mentioned in the introduction, existing research explains the lack of interest in politics and political questions in health research with the dominant position of biomedicine within the health sciences and the limited social science training health professionals receive in their medical education. Our findings challenge such generalized explanations. In fact, some of the most critical and thoughtful comments on local politics came from informants who had degrees in medicine and limited formal training in the social sciences. What seemed to matter more than educational background was long-term commitment and amount of time spent in Patria; those who had lived and worked in Patria for longer periods of time (many years) and who had close ties to the country – sometime through familial connections – were more attuned to and had a better grasp of local political dynamics than those who had not. The long-term commitment provided them with the fine-grained insight that one only can gain from spending a long time in a country. In other words, training in social sciences alone does not ensure that researchers have this kind of local knowledge.

While all the researchers acknowledged the importance of considering political context in health research, the majority were, for reasons we will discuss in the next section, reluctant to integrate questions that touched upon political issues or openly discuss the political context in Patria as part of their research. This does not mean that they were apolitical actors or disengaged from political processes. In fact, most of the researchers were situated in the applied, normative Global Health tradition [49], and expressed, implicitly or explicitly, a commitment to policy change and a desire to conduct research that would improve people’s lives or generate change through political processes. Yet, most of the researchers did not frame such engagements in political terms.

Some informants, particularly those positioned within medicine and public health, very clearly articulated that the overall aim of their research was to influence and change policy. “When you create research, you want to present it to political actors and make them, perhaps, alter their priorities somehow, and I think it’s important,” a professor in medicine and Global Health concluded. Others were more careful when describing their role as policy actors. “We don’t engage in politics as such” one of our informants explained, after emphasizing how the project’s funding agency, the Bill and Melinda Gates foundation, had explicit guidelines that prevented the research team from engaging in political activities. “We don’t try to influence policies,” he continued. “We want to present evidence, frameworks and policies, and then it is up to the decision-makers to use as they see fit.”

The way this latter interviewee frames his involvement demands further analysis. By making a distinction between politics and policy, he assumes that policy making – including the evidence and frameworks his project produce – are neutral, apolitical categories of knowledge, that exist independently of local and global power and decision-making processes. His statement, moreover, reflects a narrow definition of politics: by assuming that political power lies with national decision makers and politicians alone, he overlooks the role that powerful funding agencies, such as the Bill and Melinda Gates foundation, have in setting policy agendas [20, 38, 56]. Most importantly, as a Global Health researcher who has strong affiliations with major Global Health initiatives and institutions of higher learning in the global north, he fails to acknowledge his own political power and role as a political actor. The framing that emerges in this interview – of policy as an evidence-based, procedural tool that is distinct from politics – is common in much of public health and health policy research, where politics typically is treated as “an unwelcome ghost that causally interacts with the policy machine, disturbing rational decision-making and technical intervention” ([10], 3). This decoupling of policy from politics also legitimizes a close, unreflective collaboration between researchers and powerful local and global policy actors, allowing the researcher to operate under the pretext of scientific neutrality and a desire to “do good”. Such a framing is not necessarily deliberate but rather a reflection of entrenched, taken-for-granted discourses that dominate the health research and policy field. We could, perhaps say, that it is an example of how the anti-politics machine operates within the field of Global Health.

Informants situated within the social sciences tended to express their policy engagement in a more ambivalent manner. A professor in anthropology at a European university, who has been involved in several interdisciplinary research projects in Patria, talked about how he and his collaborators, in principle, approach their research from a critical standpoint. With a focus on politically sensitive and highly contested development schemes, the research projects that this scholar was involved in were designed as critical investigations aimed at exposing the unintended consequences of the interventions. Yet, this criticality did not prevent the research team from sharing their knowledge with concerned stakeholders, including with the Patrian government. While this researcher, and some of the other social scientists, tended to lean towards critical research, they were also self-reflective and partially questioned their relationship with the policy field. A professor in Global Health at a European university, who has a PhD degree in political science, expressed this concern as follows:

As social scientists we have a problem because we are very bad at coming up with better solutions, but we are very good at critiquing them. We are not so good at coming up with what to do, how to improve, to launch interventions that could make change. I think that is the problem when we critique things.

While most of the researchers, regardless of how they framed their policy engagement, maintained an explicit or implicit desire to improve policy, some of our informants questioned to what extent research conducted by international scholars has an impact at all. One of our interviewees, a medical doctor and professor in Global Health with long-term experience working in Patria, concluded: “Most of the papers written about health in Patria have no impact on policy whatsoever. Most of the international journal articles are written for an international audience and these papers are not read by the policy makers in Patria.” He moreover claimed that most international scholars lack proper understanding of the Patrian historical and political context and argued that to change policy and have an impact, scholars need to “learn the game.”

So, what does the game look like? As we will show below, perspectives from frame theory are analytically useful, as they can help us understand the research practices many of the scholars we interviewed employed.

### Practices of framing

To do research in Patria requires navigating a complex political landscape and it is, as one of our informants concluded, “very obvious that you should tread carefully”. To gain and maintain research access, and to protect themselves and their local collaborators in a politically sensitive and highly authoritarian context, the researchers employed both strategic and unconscious practices of framing [3, 22, 59]. Such framing practices included avoiding or leaving out terms, scholarly references, and topics that were politically loaded, strategically conforming to the assumed apolitical language and methodologies of health research, and negotiating with, leaning on, and at times hiding behind their local counterparts in processes of research dissemination and writing.

While an increasing number of international scholars do research in Patria, securing and maintaining research permits, visas and access is often a struggle. Some of the researchers we interviewed knew about or had colleagues who either had lost their research visa or research approval. A few even had colleagues who had been deported because they openly addressed politically sensitive issues. For researchers who work in authoritarian contexts, such scenarios are well known [23]. The researchers we interviewed talked a lot about the different framing practices they used to secure and maintain access to the field and to protect themselves and others. As one of our interviewees put it:

I think everyone is making somewhat rational calculated decisions on how we do research in Patria that engages these [political] questions without potentially jeopardizing someone’s career or potentially having all of this rejected based on being too forthright at the outset.

Quite a few of the researchers were involved in research projects that addressed issues that were high on the agenda of European and North American granting agencies: health related research that touched upon issues related to human rights, democracy, inclusion, and equity. At the time we conducted the interviews, laws had been passed in Patria that prohibited researchers from engaging in work related to politics, good governance, and human rights. Some of our informants described how they navigated these restrictions by strategically framing their research so that it would be acceptable in the Patrian context, avoiding politically sensitive terms, topics, and references. They would, for example, re-frame their original research proposals, developed for funding agencies, before submitting them to research ethics committees or to other institutions that provided research permits in Patria. They would leave out human rights language and use a more apolitical and less sensitive vocabulary. An anthropologist who, in collaboration with Patrian scholars, had investigated highly contentious health and development interventions, described these carefully negotiated processes:

For my Patrian colleagues there was a real risk that the framing the donor favored would make the project impossible. That it would be too risky in terms of their [his colleagues’] own reputations, in terms of possible consequences. It was certainly on my mind when we were bouncing drafts back and forth. There were some things that I was writing that might not be acceptable to them [his Patrian colleagues]. We had to come up with a compromise that would allow us to speak to the funder and make the project seem compelling, on their terms, and find a line that was acceptable on the terms of their [the Patrian colleagues’] managers and others at [a regional Patrian] University.

This quote illustrates how global donors also engage in practices of framing when they set agendas and identify priorities as conditions for research and in doing so attempt to discipline regimes. On the other hand, this comment from an anthropologist exemplifies how researchers respond to, negotiate, and re-frame their research, particularly when political realities on the ground do not align with donor language and commitments.

In addition to re-framing research proposals and avoiding terms that were politically loaded, some of the researchers also talked about how they avoided referencing certain authors, organizations, or policy actors who have been critical of the regime:

The Patrian PI attended very closely to whom we were citing. So, it was not so much about what we wrote, as who we cited as authorities. Citing some of the activist organizations such as the Oakland Institute and Human Rights Watch in particular, was a reach too far. As I recall we were able to maintain the framing, but we removed citations to those organizations’ reports. We either left some of our claims unsupported by citations or found appropriate and more academic references.

Some of the researchers also took advantage of and leaned on the assumed apolitical nature of health research. An anthropologist with a public health background described how he was very careful about how he presented himself and his research interests. He specifically mentioned that other anthropologists he knew had burned bridges to local authorities by being too forthright. To avoid raising red flags, he presented himself as a health researcher, rather than an anthropologist. Another social scientist talked about how he secured local ethical approval by conforming to the perceived apolitical methodologies of health research. By emphasizing the research process and structure, he managed to “skirt around” the fact that his research was rather political in nature.

I presented it as a kind of household survey, and so it looked kind of typical, it did not look so much focused on policy per se (…) I did not outline what the findings could potentially be, it was more just: “here’s the process”. So, you know “we use interviews, discussions, focus groups, we’re using this co-production methodology” and so on. And so, they assessed it based on its structure, and I just did not open the door of where this might go in terms of asking about policies that benefit the relatively wealthy and exclude the poor.

For some of the scholars, these framing practices enabled them to address political issues, albeit in a more subtle manner. While they adopted framing practices that made their research appear apolitical, they still paid attention to, and incorporated political issues into their work, including in their publications. We could say that they were engaged in a subtle, low-profile and undeclared form of resistance – what James Scott terms infra-politics [53]– that allowed them to not only work constructively within but also challenge the dominant system of power. Others, however, were much more reluctant to address politically sensitive issues, or to engage in a critique – overtly or covertly – of the regime. They described how they purposefully left out issues that were too political or pushed findings that suggested that political factors had an influence on certain health outcomes, “into the background”. They argued that such strategies allowed them to conduct meaningful work, influence policy, and reach their intended goals. Maintaining access to the field was a key issue. One of our interviewees detailed how she had developed trusting relationships with people at local universities and government institutions. For her, maintaining that hard fought access legitimized leaving out politically sensitive issues:

I would never do research on anything to jeopardize that relationship. I have been working for almost ten years now. They all know who I am, they all love my work, they think it is important, they are willing to give me facilities, and vehicles sometimes, and access. So, there are certain things I will never talk about, like cholera. I cannot do a research project on cholera even though I really want to because it is threatening. And my interlocutors and my key informants, my contacts at [name of transnational organization], the community health workers, nurses – they cannot talk about cholera. So, it endangers their work, and you know I could never have research assistants asking questions about cholera. That would just be unheard of, so it is great to have that kind of access, but I realize that it is not a blank check.

While some of the researchers we interviewed defended their avoidance practices, others worried about the potential biases such practices generated. An epidemiologist from a European university critically reflected on how the exclusion of certain topics prevented her research team from researching important risk factors:

When we are looking at risk factors [referring to a specific health problem] we do not put ethnicity or religion into the multivariable model. That is very deliberate because it is such a sensitive area to go down. If you ask a question about religion in [name of town], you will probably find that being Muslim is associated with lower health. (…) We feel that there would be a very sort of strong rationale for looking at ethnicity per se and it being a proxy for other social disadvantages. But of course, that [leaving ethnicity out of the model] kind of wipes ethnicity away, doesn’t it?

While we, in our research, primarily focused on the way international researchers navigated politics, many of the researchers we interviewed emphasized the crucial role their Patrian collaborators played in these framing processes. One of our interviewees detailed how her Patrian partner, from a local research organization, was hesitant to publish a politically charged finding. Their research had revealed that community health workers (CHW) often were mobilized for political purposes. Political leaders would, for example, ask the CHWs to make public statements in support of political campaigns. This researcher argued that such engagements negatively impacted the health workers’ credibility in the community. “I really wanted to include this in the paper, but our colleagues in our local partner organization were very hesitant. I finally convinced them to include it, but with some nuance”.

While this researcher pushed and negotiated with her Patrian counterpart to publish a finding that could be read as a critique of the country’s health care system, others were more cautious. They maintained that their Patrian partners knew best how to frame difficult issues – determining how to formulate research questions and proposals, what to publish and what to withhold – and had greater leverage in influencing policy. “We depend a lot on our Patrian partners to inform our thinking and critically review our writing”, a professor in Global Health at a European university explained. She recounted how a Patrian co-author had removed several sentences from an article one of her students had drafted, saying “This is too much”. “We immediately removed it”, the professor concluded, emphasizing how the research team was concerned their research, which focused on reproductive health and rights, could potentially cause harm.

Several of our informants highlighted how they relied on their Patrian colleagues in conveying their research to policy makers. One medical doctor and professor in Global Health, who had mentored many Patrian PhD students, talked about how he would leave it up to his students to convey research findings to the government. This was, according to him, the only way his research could have an impact on policy:

I have lived in Patria for many years, so I know the game, but I could not change the policy. But my Patrian PhD students, they had the right context, they attended the right political meetings, they knew how to make influence. So, they presented their research findings, they are pure professionals. Then the government said, ‘this is interesting’. Your evaluations show that we are not doing good on tuberculosis, so we will try this. So, the government changed their policy.

To work closely with local counterparts is an important and well-known strategy for scholars who conduct research in authoritarian contexts [23]. As some of our interviewees emphasized, in many cases, Patrian scholars know better how to navigate local politics and how to best convey research findings to relevant policy makers. However, they are also more likely to suffer the consequences of critical research. To unconditionally lean on local counterparts to make decisions about acceptable and strategic ways to convey research, is therefore not without its challenges. It is an act of framing that functions as a disavowal of responsibility. If we assume that the overall aim we have is to ensure that “people come first” [8], one could argue that we have an ethical obligation to speak truth to power where others are not able, and to reveal weaknesses and challenge dominant political narratives, even if it could jeopardize our research. The fact that we as Northern Global Health scholars are in privileged positions due to our citizenship in powerful Western donor countries and protected from the political consequences that our local counterparts may face leave us with considerable ethical responsibility.

## Concluding discussion

By interviewing Northern Global Health scholars who engage in health research in the context of an authoritarian state, we have nuanced the broad-brush portrayal of Global Health scholars as disinterested in or uninformed about the intersection of health and politics. While some of our interviewees indeed framed their Global Health research engagement in apolitical ways, all the researchers we interviewed, regardless of educational background, recognized health as a political matter and acknowledged the importance of considering politics and political context in health research. Yet, the majority of our interlocutors were reluctant to explicitly integrate politically sensitive topics and discuss questions related to the local political context in their research and publications. The researchers we interviewed employed various practices of framing, including positioning themselves within the language and methodologies of a perceived apolitical health field, leaning on their local counterparts, and avoiding politically loaded language, questions and references. While some of these framing practices were motivated by an interest in influencing and changing policy, they were also closely linked to, and spurred by, a desire to gain and maintain access to the field, protect themselves and their Patrian collaborators, and avoid political complications getting in the way of their research.

How do we make sense of these framing practices? Are they problematic? What potential unintended consequences and effects do they pose for healthcare research? In the final paragraphs of our paper, we examine these questions. First, we discuss practices of framing in light of the literature on fieldwork in authoritarian settings. In particular, we explore the potential unintended consequences these practices could have for the broader field of healthcare research. Second, we discuss the value of a turn to political reflexivity in the healthcare sciences.

### Framing as a research strategy in authoritarian contexts – and in the healthcare sciences

It is important to acknowledge that the practices of framing we here have detailed are not unique. They are thoroughly discussed, defended *and* recommended in the emerging social-science literature on fieldwork and authoritarianism [2, 23, 38, 41]. In a meta-analysis of this literature, David Art provides a synthesis of ‘best practices’ for research in authoritarian settings, specifically suggesting that we should “frame the research topic in a way that has the best chance of reducing any sensitivity around it” ([2], 980). Political scientists and anthropologists who conduct research in authoritarian settings maintain that although they “adapt their wording and behavior to remain within the red lines” [23] they are nevertheless able to produce meaningful and “excellent and informed scholarship” ([41], 924). Such arguments are legitimate and well-founded. Yet, we caution against an *uncritical* adoption of these practices in healthcare research. We do so because fundamental differences exist between social-science research that explicitly is attuned to the study of political systems and authoritarian regimes, and healthcare research. First, scholars who discuss and recommend practices of framing as a depoliticization strategy for research conducted in authoritarian settings, use these tactics to better understand the political dynamics and practices in their respective research settings. Rather than avoiding politics – which is common practice in most healthcare research – they find alternative ways to collect data and conduct research on political systems and practices of power. In other words, they explicitly study and are very attentive to politics, albeit in a covert manner. Secondly, the research anthropologists and social scientists conduct on authoritarianism and in authoritarian settings, tends to be rather *descriptive and interpretive* in nature. In contrast, the majority of scholars within the field of health have *normative* aspirations, and “focus on improving the health of specific populations by applying novel interventions and measuring the successes thereof” ([9], 2). The normative, applied and political nature of healthcare research requires that we are cautious and reflexive about the potential unintended consequences and effects our framing could have in practice. What biases do our framing practices produce when we leave out certain questions or indicators, or avoid researching a specific disease? What effects and unintended consequences do these biases have on healthy policy making and health systems? Do our framing practices exacerbate social and economic inequalities? Does our engagement in policy making legitimize authoritarian regimes? By asking these questions, our intention is not to condemn practices of framing in healthcare research. But, given the applied and normative nature of the healthcare sciences, our point is rather to highlight how important it is that we are open about the potential unintended and political consequences our framing practices may have on the quality of our research. In other words, we call for more political reflexivity.

### Towards political reflexivity in global health

In recent years, scholars have increasingly emphasized the importance of reflexivity in healthcare research ([9, 45, 52], 1). In addition to encouraging transparency on all aspects of the research, this body of scholarship highlights the value of critically discussing inequities and power imbalances in research relationships [52], and questions related to researcher identity and positionality [39]. We welcome this turn to reflexivity and the increased focus on openness, including discussions about inequities and power relations in research partnerships. We would, however, like to emphasize that what counts as reflexivity is not given. While “structured reflexivity statements” [44] have the potential to capture important aspects of our research partnerships, we caution against the ‘one-size-fits-all’ nature of such solutions. As anthropologist Susan Wright has argued, it is particularly important that we are “self-critical about the way we deploy key words such as reflexivity, and with what effects” ([61], 145). Here, health science researchers can learn much from anthropology, where discussions about reflexivity in knowledge production have been ongoing since the 1970s. Anthropologists have, for example, cautioned against a “stunted” and “inward” approach to reflexivity that fails to examine the broader political and institutional environment in which our research is conducted [61]. Judith Okley ([48], 3) even argues that “a reflexivity which excludes the political is itself unreflective”.

We suggest, that if the turn to reflexivity in healthcare research is to be meaningful, it requires a commitment to and engagement with questions related to politics and local political context. To simply acknowledge that health is political and that politics matter – as all our interviewees did – does not suffice. Nor do reflexivity statements. Political reflexivity necessitates a certain level of political literacy: an ability to critically discuss, recognize and examine how history, political culture and practices of power impact population health and health systems, and interact with our research. It moreover entails studying how paradigm shifts in public health policy brought by new political regimes may impact the work and careers of researchers, especially those who are relatively marginalized and vulnerable. Political reflexivity also demands transparency – a willingness to examine and discuss our own political and philosophical leanings as potential biases. It is therefore pertinent that we as healthcare researchers know the local power dynamics and the political context in which we conduct our research [45]. This is particularly important for research conducted in authoritarian settings where the political situation often is unpredictable, where certain events or dynamics may be taboo or unstated [41], and where social desirability bias is heightened due to limited freedom of speech. In such contexts, people are not free to speak their minds, and often reproduce the official story. It is critical that we as healthcare researchers understand why and under what circumstances self-censorship and social-desirability bias are likely to emerge. The increase in authoritarianism in our contemporary world – even in places and contexts we wrongly may have thought have systems and institutions in place that will prevent it from surfacing – makes it even more pertinent that we know and recognize the symptoms of authoritarianism.

While training in the social sciences – in fields such as history, anthropology and political science – may provide health science researchers with knowledge and analytical tools that facilitate critical reflection and awareness around these issues, it does not necessarily provide the fine-grained, experiential knowledge that we need in order to be able to differentiate between the official and unofficial discourse – crucial for assessing data validity and reliability – in contexts such as Patria. This is knowledge that is conditioned on our ability to establish trusting relationships. It is an embodied form of expertise that is born out of curiosity and of close interactions with people on the ground. It is a form of expertise that we cannot gain from short-term, “parachute research.”

The political reflexivity we argue for here is not set in stone, nor is it about showing compliance with a set of predetermined ideals for what is considered to be ethical and equitable research. The political reflexivity we are proposing is both an ideal – something to strive for – as well as an attitude. It is a dual reflexivity characterized by inward and outward openness: an inward openness that allows us to critically explore, think, and write as honest as we can about what we do and experience as health researchers; and an outward openness to explore the complex political environments and broader contexts in which we conduct our research. The political reflexivity we here suggest can help us reflect upon the taken-for-granted presuppositions and norms that guide our research, and better understand how political environments and power dynamics shape and are shaped by our research.

## Data Availability

The data that supports the findings of this study is not publicly accessible but is available from the corresponding author upon reasonable request.

## References

[1] Abimbola S. The foreign gaze: authorship in academic global health. BMJ Specialist Journals. 2019. 10.1136/bmjgh-2019-002068 PMC6830280 31750005

[2] Art D. Archivists and adventurers: research strategies for authoritarian regimes of the past and present. Soc Sci Q. 2016;97(4):974–90.

[3] Bacchi C. 2009. "The Issue of Intentionality in Frame Theory. The Need for Reflexive Framing." In The Discursive Politics of Gender Equality, edited by E. Lombardo, P. Meier and M. Verloo. London: Routledge/ECPR Studies in European Political Science.

[4] Bacchi C. Strategic interventions and ontological politics: research as political practice. In Engaging with Carol Bacchi. Strategic interventions and exchanges, edited by Angelique Bletsas and Chris Beasley. Adeleide: University of Adeleide Press. 2012:141–156.

[5] Bacchi C. Problematizations in health policy: questioning how “problems” are constituted in policies. SAGE Open. 2016;6(2): 2158244016653986.

[6] Bateson G. Steps to an ecology of mind: collected essays in anthropology, psychiatry, evolution, and epistemology. University of Chicago press; 2000.

[7] Benford RD, Snow DA. Framing processes and social movements: an overview and assessment. Ann Rev Sociol. 2000;26(1):611–39.

[8] Biel J, Petryna A, editors. When people come first. Critical studies in global health. Oxford: Princeton University Press; 2013.

[9] Borst RAJ, Wehrens R, Bal R. “And when will you install the new water pump?”: disconcerted reflections on how to be a ‘good’ global health scholar. Glob Health. 2023;19(1):19. 10.1186/s12992-023-00919-8. 10.1186/s12992-023-00919-8 PMC10029300 36944977

[10] Bruen C, Brugha R. A ghost in the machine? Politics in global health policy. Int J Health Policy Manag. 2014;3(1):1–4. 10.15171/ijhpm.2014.59. 24987713 10.15171/ijhpm.2014.59 PMC4075096

[11] Costa N, Blyth FM, Parambath S, Schneider CH. What’s the low back pain problem represented to be? An analysis of discourse of the Australian policy directives. Disabil Rehabil. 2023;45(20):3312–22. 10.1080/09638288.2022.2125085. 36150033 10.1080/09638288.2022.2125085

[12] D’Angelo P. Studying framing in political communication with an integrative approach. Am Behav Sci. 2012;56(3):353–64. 10.1177/0002764211426332.

[13] Danell J-A, Jarl J. What is the problem represented to be in the Swedish police authority: a policy analysis. Policing A J Policy Pract. 2024;18: paae041. 10.1093/police/paae041.

[14] Davies S. Global politics of health. Cambridge, UK: Polity; 2010.

[15] Dawes DE. The political determinants of health. Baltimore: Johns Hopkins University Press; 2020.

[16] De Kock C. Cultural competence and derivatives in substance use treatment for migrants and ethnic minorities: what’s the problem represented to be?" Social Theory & Health. 10.1057/s41285-019-00113-0. 2019.

[17] Dean M. Governmentality: power and rule in modern society. London: Sage; 1990.

[18] Dee EC, Eala MA, Robredo JP, Ramiah D, Hubbard A, Ho FD, Sullivan R, Aggarwal A, Booth CM, Legaspi GD, Nguyen PL. Leveraging national and global political determinants of health to promote equity in cancer care. JNCI J Natl Cancer Inst. 2023;115(10):1157–63. 37402623 10.1093/jnci/djad123 PMC10560599

[19] Doyal L, Pennel I. The political economy of health. London: Pluto Classic; 1979.

[20] Fejerskov AM. The Gates Foundation's rise to power: private authority in global politics. Abingdon, Oxon: Routledge; 2018.

[21] Ferguson J. The anti-politics machine: “Development”, depoliticization and bureaucratic power in Lesotho. Cambridge: Cambridge University Press; 1990.

[22] Ferree MM. Inequality, intersectionality and the politics of discourse. Framing feminist alliances." In the discursive politics of gender equality. Streching, bending and policymaking, edited by Emanuela Lombardo, Petra Meier and Mieke Verloo, 86-104. Oxcon: Routledge/ECPR Studies in European Political Science. 2009.

[23] Glasius M, De Lange M, Bartman J, Dalmasso E, Lv A, Del Sordi A, Michaelsen M, Ruijgrok K. Research, ethics and risk in the authoritarian field. Cham: Springer Nature; 2018.

[24] Goffman E. Frame analysis: an essay on the organization of experience. Cambridge: Harvard University Press; 1974.

[25] Gomez E. Introduction: the state of political science research in global health politics and policy. Global Health Governance. 2016;10(3):3–8.

[26] Goode JP, Ariel IA. Special Issue Guest Editors’ Introduction: observing autocracies from the ground floor. Soc Sci Quart. 2016;97(4):823–33.

[27] Gore R, Parker R. Analysing power and politics in health policies and systems. Glob Public Health. 2019;14(4):481–8. 10.1080/17441692.2019.1575446. 30773135 10.1080/17441692.2019.1575446

[28] Gruenbaum E. Medical anthropology, health policy and the state: a case study of Sudan. Policy Stud Rev. 1981;1:47–65. 11632733 10.1111/j.1541-1338.1981.tb00377.x

[29] Hafferty FW, Franks R. The hidden curriculum, ethics teaching, and the structure of medical education. Acad Med. 1994;69(11):861–71. 10.1097/00001888-199411000-00001. 7945681 10.1097/00001888-199411000-00001

[30] Harper I, Parker M. The politics and anti-politics of infectious disease control. Med Anthropol. 2014;33(3):198–205. 10.1080/01459740.2014.892484. 24761974 10.1080/01459740.2014.892484

[31] Jasanoff S, Hilgartner S, Hurlbut JB, Özgöde O, Rayzberg M. Comparative Covid response: crisis, knowledge, politics. Ithaca: CompCoRe Network, Cornell University; 2021.

[32] Keck ME, Sikkink K. Activists beyond borders: advocacy networks in international politics. New York: Cornell University Press; 1998.

[33] Kenworthy NJ, Richard P. HIV scale-up and the politics of global health. Glob Public Health. 9(1–2):1–6. https://www.tandfonline.com/doi/abs/10.1080/17441692.2014.880727. 10.1080/17441692.2014.880727 24555669

[34] Kickbusch I. The political determinants of health–10 years on. BMJ. 2015;350: h81. 10.1136/bmj.h81. 25569203 10.1136/bmj.h81

[35] Koon AD, Hawkins B, Mayhew SH. Framing and the health policy process: a scoping review. Health Policy Plan. 2016;31(6):801–16. 26873903 10.1093/heapol/czv128 PMC4916318

[36] Koon AD, Mendenhall E, Eich L, Adams A, Borus ZA. A spectrum of (Dis)Belief: coronavirus frames in a rural midwestern town in the United States. Soc Sci Med. 2021;272: 113743. 10.1016/j.socscimed.2021.113743. 33592395 10.1016/j.socscimed.2021.113743 PMC8723978

[37] Lee K. Revealing power in truth: comment on “Knowledge, moral claims and the exercise of power in global health.” Int J Health Policy Manag. 2015;4(4):257–9. 10.15171/ijhpm.2015.42. 25844390 10.15171/ijhpm.2015.42 PMC4380571

[38] Levich J. The gates foundation, ebola, and global health imperialism. Am J Econ Sociol. 2015;74(4):704–42. 10.1111/ajes.12110.

[39] Liwanag HJ, Rhule E. Dialogical reflexivity towards collective action to transform global health. BMJ Glob Health. 2021;6(8): e006825. 10.1136/bmjgh-2021-006825. 34417275 10.1136/bmjgh-2021-006825 PMC8381326

[40] Lombardo E, Meier P. Framing gender equality in the European Union political discourse. Soc Polit. 2008;15(1):101–29. 10.1093/Sp/Jxn001.

[41] Loyle CE. Overcoming research obstacles in hybrid regimes: lessons from Rwanda*. Soc Sci Q. 2016;97(4):923–35. 10.1111/ssqu.12346.

[42] McCoy D, Singh G. A spanner in the works? anti-politics in global health policy: comment on “A ghost in the machine? politics in global health policy.” Int J Health Policy Manag. 2014;3(3):151–3. 10.15171/ijhpm.2014.77. 25197681 10.15171/ijhpm.2014.77 PMC4154554

[43] Moon S. Power in global governance: an expanded typology from global health. Glob Health. 2019;15(1):1–9. 10.1186/s12992-019-0515-5 PMC6881906 31775779

[44] Morton B, Vercueil A, Masekela R, Heinz E, Reimer L, Sepeedeh Saleh C, Kalinga MS, Biccard B, Chakaya J. Consensus statement on measures to promote equitable authorship in the publication of research from international partnerships. Anaesthesia. 2022;77(3):264–76. 34647323 10.1111/anae.15597 PMC9293237

[45] Naidu, Thirusha, Gareth Gingell, and Zareen Zaidi. 2024. "Decolonial framework for applying reflexivity and positionality in global health research." *Global Health Promotion* 0 (0):17579759241238016. 10.1177/17579759241238016. 10.1177/17579759241238016 PMC11363464 38566278

[46] Namugumya BS, Candel JJL, Termeer CJAM, Talsma EF. The framing of malnutrition by parliamentarians in Uganda. Health Policy Plan. 2021;36(5):585–93. 10.1093/heapol/czab009. 33709155 10.1093/heapol/czab009

[47] Navarro V. Politics and health: a neglected area of research. Eur J Pub Health. 2008;18(4):354–5. 10.1093/eurpub/ckn040. 18524802 10.1093/eurpub/ckn040

[48] Okely J. Anthropology and autobiography. Participatory experience and embodied knowledge. In: Anthropology and autobiography. Routledge: London; 1992. p. 13–40.

[49] Ooms G. Navigating between stealth advocacy and unconscious dogmatism: the challenge of researching the norms, politics and power of global health. Int J Health Policy Manag. 2015;4(10):641–4. 10.15171/ijhpm.2015.116. 26673173 10.15171/ijhpm.2015.116 PMC4594103

[50] Pienaar K, Murphy DA, Race K, Lea T. Problematising LGBTIQ drug use, governing sexuality and gender: a critical analysis of LGBTIQ health policy in Australia. Int J Drug Policy. 2018;55:187–94. 29395699 10.1016/j.drugpo.2018.01.008

[51] Rae J, Green B. Portraying reflexivity in health services research. Qual Health Res. 2016;26(11):1543–9. 26935721 10.1177/1049732316634046

[52] Saleh, Sepeedeh, Refiloe Masekela, Eva Heinz, Seye Abimbola, Equitable Authorship Consensus Statement Group, Ben Morton, Andre Vercueil, Lisa Reimer, Chisomo Kalinga, and Maaike Seekles. Equity in global health research: a proposal to adopt author reflexivity statements. PLOS global public health. 2022;2(3): e0000160. 36962165 10.1371/journal.pgph.0000160 PMC10022150

[53] Scott, James C. 2012. Infrapolitics and Mobilizations: A Response by James C. Scott 1. *Revue française d’études américaines* (1):112–117.

[54] Shiffman J, Shawar YR. Framing and the formation of global health priorities. Lancet. 2022;399(10339):1977–90. 10.1016/S0140-6736(22)00584-0. 35594874 10.1016/S0140-6736(22)00584-0

[55] Singer M. The coming of age of critical medical anthropology. Soc Sci Med. 1989;28(11):1193–203. 10.1016/0277-9536(89)90012-9. 2660276 10.1016/0277-9536(89)90012-9

[56] Storeng KT. The GAVI Alliance and the “Gates approach” to health system strengthening. Glob Public Health. 2014;9(8):865–79. 10.1080/17441692.2014.940362. 25156323 10.1080/17441692.2014.940362 PMC4166931

[57] Storeng KT, Abimbola S, Balabanova D, McCoy D, Ridde V, Filippi V, Roalkvam S, Akello G, Parker M, Palmer J. Action to protect the independence and integrity of global health research. BMJ Glob Health. 2019;4(3): e001746. 10.1136/bmjgh-2019-001746. 31297249 10.1136/bmjgh-2019-001746 PMC6590965

[58] Der Haar V, Marleen, M Verloo. Starting a conversation about critical frame analysis: reflections on dealing with methodology in feminist research. Polit Gend. 2016;12(3):E9.

[59] Van Hulst M, Dvora Y. 2016. "From policy “frames” to “framing” theorizing a more dynamic, political approach." The American review of public administration 46 (1):92-112.

[60] Verloo MMT. Mainstreaming gender equality in Europe: a critical frame analysis approach. 2005.

[61] Wright S. Politically reflexive practitioners. Curr Policies Pract Eur Soc Anthropol Educ. 2004;2:34–52.

[62] Yusupova G. Exploring sensitive topics in an authoritarian context: an insider perspective*. Soc Sci Q. 2019;100(4):1459–78. 10.1111/ssqu.12642.

